# Optimization of cAMP fluorescence dataset from ACTOne cannabinoid receptor 1 cell line

**DOI:** 10.1016/j.dib.2016.03.086

**Published:** 2016-04-01

**Authors:** Chaela S. Presley, Ammaar H. Abidi, Bob M. Moore

**Affiliations:** University of Tennessee Health Science Center, College of Pharmacy, United States

**Keywords:** ACTOne, Cannabinoids, CB1, Pharmacology, cAMP

## Abstract

The ACTOne cannabinoid receptor 1 functional system is comprised of transfected HEK cells with the parental cyclic nucleotide gated channel (CNG) co-transfected with cannabinoid receptor 1 (CB1). The ACTOne CB1 cell line was evaluated for cAMP driven fluorescence by optimizing experimental conditions for sensitivity to forskolin and CP 55,940, reading time point, reliability of cell passage number, and pertussis inactivation of G_i/o_.

## Specifications table

**Table:** 

Subject area	Biology
More specific subject area	Pharmacology
Type of data	Graph
How data was acquired	Biotek Synergy 2 Plate reader
Data format	Raw fluorescent values and normalized values
Experimental factors	Forskolin concentration, cell passage number, reading time point, pertussis concentration
Experimental features	HEK cells with CNG+CB1 receptor were optimized for evaluating ligand G_i/o_ and G_s_ pharmacology at the CB1 receptor
Data source location	Memphis, TN, USA
Data accessibility	Data in the article

## Value of the data

• These data establish a new functional cell line that evaluates putative CB1 ligands for pharmacologic activity.
• The fluorescent detection of cAMP changes allows for a high throughput method of detection that can then be compared to the parental cell line to determine off-target influences.
• This system can also be pretreated with pertussis toxin to reveal CB1 G_s_ coupling specific to cannabinoid ligands.

## 1 Data

The ACTOne CB1 cell line was optimized for experimental conditions related to 96 well plate reader detection of cAMP driven fluorescence. Cells were probed for optimal forskolin stimulation of cAMP driven fluorescence, the concentration of CB1 agonist CP 55,940 needed to suppress cAMP driven fluorescence, as well as the time point for most stable fluorescent signal. Furthermore, the robustness of the ACTOne CB1 cells was evaluated with respect to cell passage number and the ability of the cell line to reveal G_s_ coupled responses following pertussis toxin G_i/o_ inactivation.

## 2 Experimental design, materials and methods

### 2.1 Plating conditions

Cells were plated in DMEM with 10% FBS and 1% P/S at 50,000 cells/100 µL well in 96 well poly-d-lysine plates and left overnight at 37 °C, 5% CO_2_. Prior to testing, 100 µL of Membrane Potential dye was added to each well, then the plate was then incubated for 1 hour in the dark at room temperature. Plates were read (Ex 540 nm, Em 590 nm) at baseline, and then after addition of compounds plates were read for 60 min.

### 2.2 Forskolin and CP 55,490 optimization

**Fig. 1:**
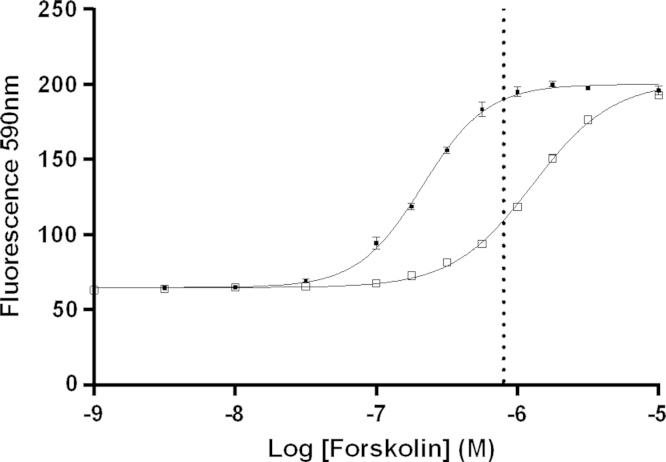
Forskolin effects in ACTOne CB1 cells (black circles) and the suppression by CP 55,940 (white squares), *N*=6, error is SEM. Vertical line is 800 nM forskolin.

ACTOne HEK-CNG+CB1 cells were evaluated for optimal sensitivity to forskolin driven cAMP fluorescent signal by exposing the cells to increasing concentrations of forskolin from 10^−4^ to 10^−9^ M (Fig. 1). Fluorescent signal plateau began at 800 nM forskolin, and suppression of forskolin signal using CB1 agonist CP 55,940 at 1 µM resulted in a significant dextral shift in the EC_50_ (212.8 vs. 1284 nM, *p*<0.0001) (Fig. 1).

**Fig. 2:**
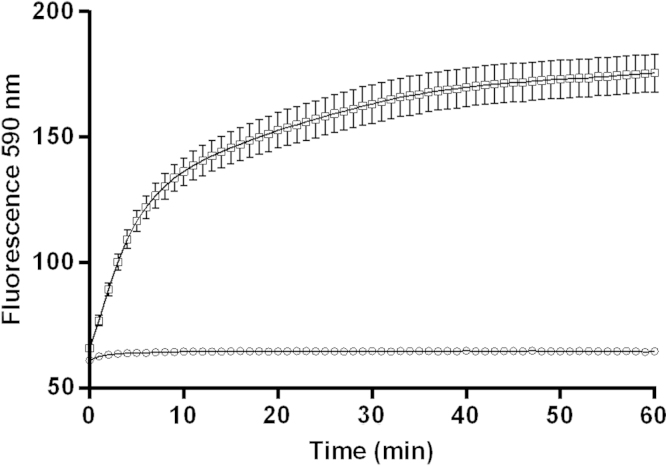
Kinetic evaluation of cAMP driven fluorescent signal at 1 min intervals in ACTOne CB1 cells with no forskolin (baseline – white circles) and 800 nM forskolin (white squares). *N*=20, error is SEM.

Evaluation of the optimal time point for fluorescent signal was determined to not be significantly different from 30 to 60 min, but a time point of 50 min was appropriate for technical efficiency and coincided with manufacturer׳s recommendations (Fig. 2) [1]. Baseline did not change during the incubation.

**Fig. 3:**
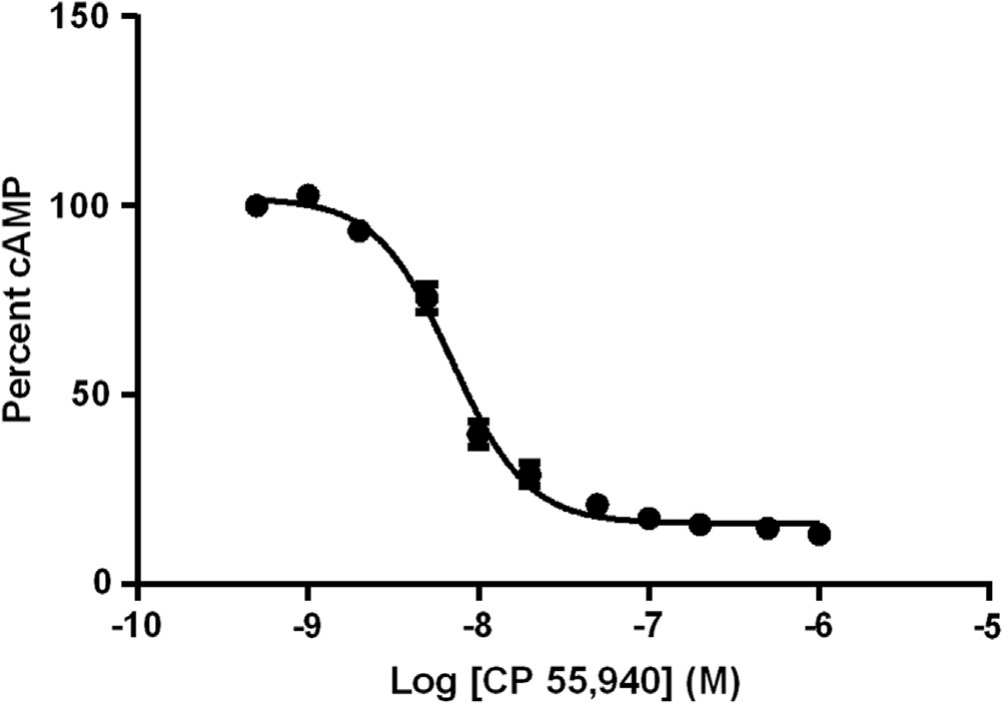
Normalized signal cAMP driven fluorescence of CP 55,490 in ACTOne CB1 cells from passage 3 to 36. *N*=26 from all control runs in [2] with EC_50_ 7.00±1.2 nM, error bars are SEM.

Response of ACTOne CB1 cells was normalized to forskolin as maximum signal and a blank baseline as minimum signal. The normalized response of ACTOne CB1 cells was determined to be robust and reproducible with respect to CP 55,490 suppression of cAMP from passages 3–36 (Fig. 3).

### 2.3 Pertussis toxin optimization

**Fig. 4:**
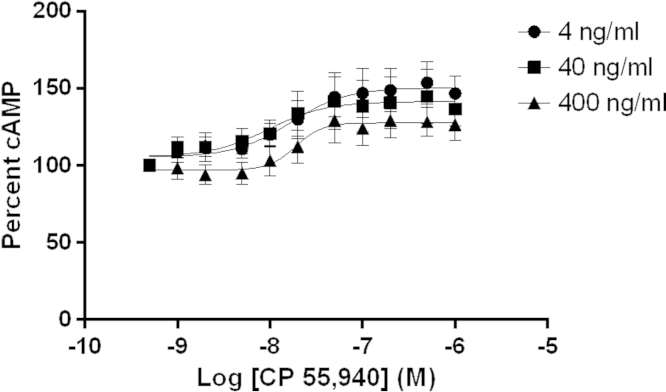
Pertussis inactivation of G_i/o_ in ACTOne CB1 cells reveals G_s_ coupling. *N*=6, error is SEM.

**Fig. 5:**
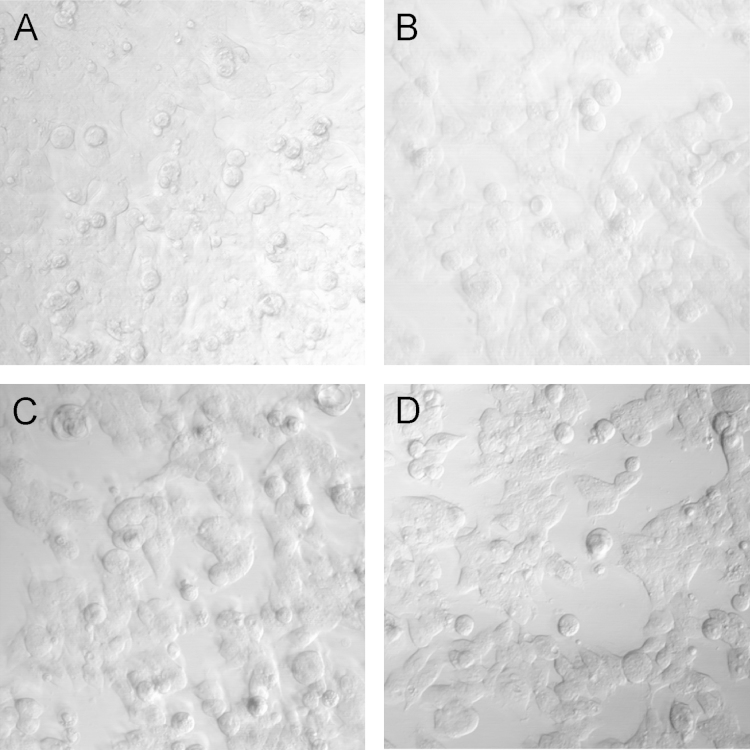
Overnight treatment of ACTOne CB1 cells with PTx: (A) 0 ng/mL, (B) 4 ng/mL, (C) 40 ng/mL, and (D) 400 ng/mL revealed lower cell confluence with higher concentrations.

The optimal pertussis toxin (PTx) dose of 4 ng/mL was determined to reveal CB1 G_s_ coupling associated with CP 55,940 (Fig. 4). While increasing concentrations of PTx did not result in statistically significant changes in cAMP driven fluorescence, signal was lower at 400 ng/mL and visual inspection revealed lower cell confluence at higher PTx concentrations (Fig. 5).

### 2.4 Kinetic evaluation

Experiments with ACTOne CB1 cells were run for 60 min and cAMP signals were evaluated at one minute intervals. Codex Biosolutions recommended reading fluorescence at 50 min [1]. Fluorescent signal was not significantly different from 60 min (*p*=0.792, *t*-test).

### 2.5 Normalization

ACTOne assay data were normalized using a feature scaling equation:

$$X^{\prime }=A\frac{(X-X_{\min})\left(B-A\right)}{X_{\max}-X_{\min}}$$

where forskolin only stimulation is the experimental maximum *B*, a blank well with dye and cells only is the experimental minimum *A*, normalized value is *X*ʹ, *X_max_* is 100%, and *X_min_* is 0%. Data were then analyzed using non-linear regression analysis.

### 2.6 Pertussis Inactivation studies

Pertussis (PTx) dose response was performed in ACTOne CB1 cells with 0, 4, 40, or 400 ng/mL (Fig. 4). Cells were incubated overnight with PTx and run against CP 55,940 the next day. Visual inspected indicated that higher concentrations of PTx resulted in lower cell attachment to plates (Fig. 5). 4 ng/mL of PTx resulted in the strongest CP 55,940 G_s_ effect on cAMP while giving the best cellular confluence.
